# Supplementary material for: Pervasive suppressors halt the spread of selfish Segregation Distorter in a natural population
Source: bioRxiv. 2025 Oct 14:2025.10.13.681989. Preprint. [Version 1] doi: 10.1101/2025.10.13.681989 (PMC12633045; doi:10.1101/2025.10.13.681989)
Supplement: 1 [file NIHPP2025.10.13.681989v1-supplement-1.pdf]

bioRxiv preprint doi: https://doi.org/10.1101/2025.10.13.681989; this version posted October 14, 2025. The copyright holder for this preprint (which was not certified by peer review) is the author/funder, who has granted bioRxiv a license to display the preprint in perpetuity. It is made available under aCC-BY 4.0 International license.

Supplementary Figures

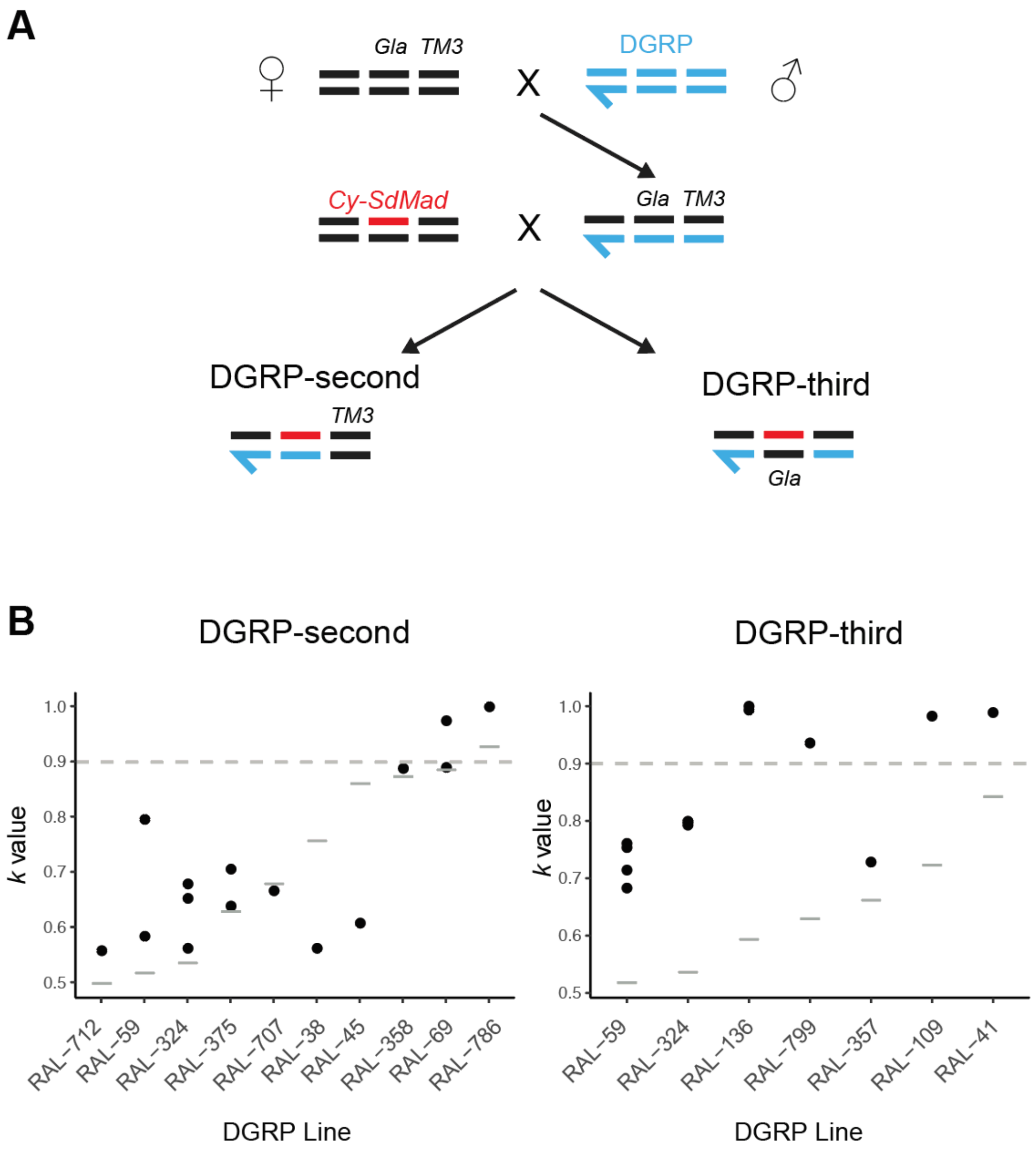

Fig S1. Surveys of dominant suppressors or intermediate sensitive *Rsp* on individual autosomes form DGRP. (A) Crossing scheme to introgress DGRP second or third chromosomes into a standard SD background. Males from each DGRP line were crossed to *Gla TM3* females, and F1 progeny carrying *Gla TM3* were crossed to *Cy-SdMad* tester flies to generate DGRP second or third chromosomes in a common *SD* background. **(B)** Variation in drive strength (*k* values) among DGRP lines for the second (left) and third (right) chromosomes. Black points indicate individual replicate measurements, and gray bars represent *k* from the "No X suppressors" crosses, indicating the combination of effects from all autosomes. Other than RAL-786, each DGRP strain tested here carries autosomes with dominant suppressors from previous surveys.

bioRxiv preprint doi: https://doi.org/10.1101/2025.10.13.681989; this version posted October 14, 2025. The copyright holder for this preprint (which was not certified by peer review) is the author/funder, who has granted bioRxiv a license to display the preprint in perpetuity. It is made available under aCC-BY 4.0 International license.

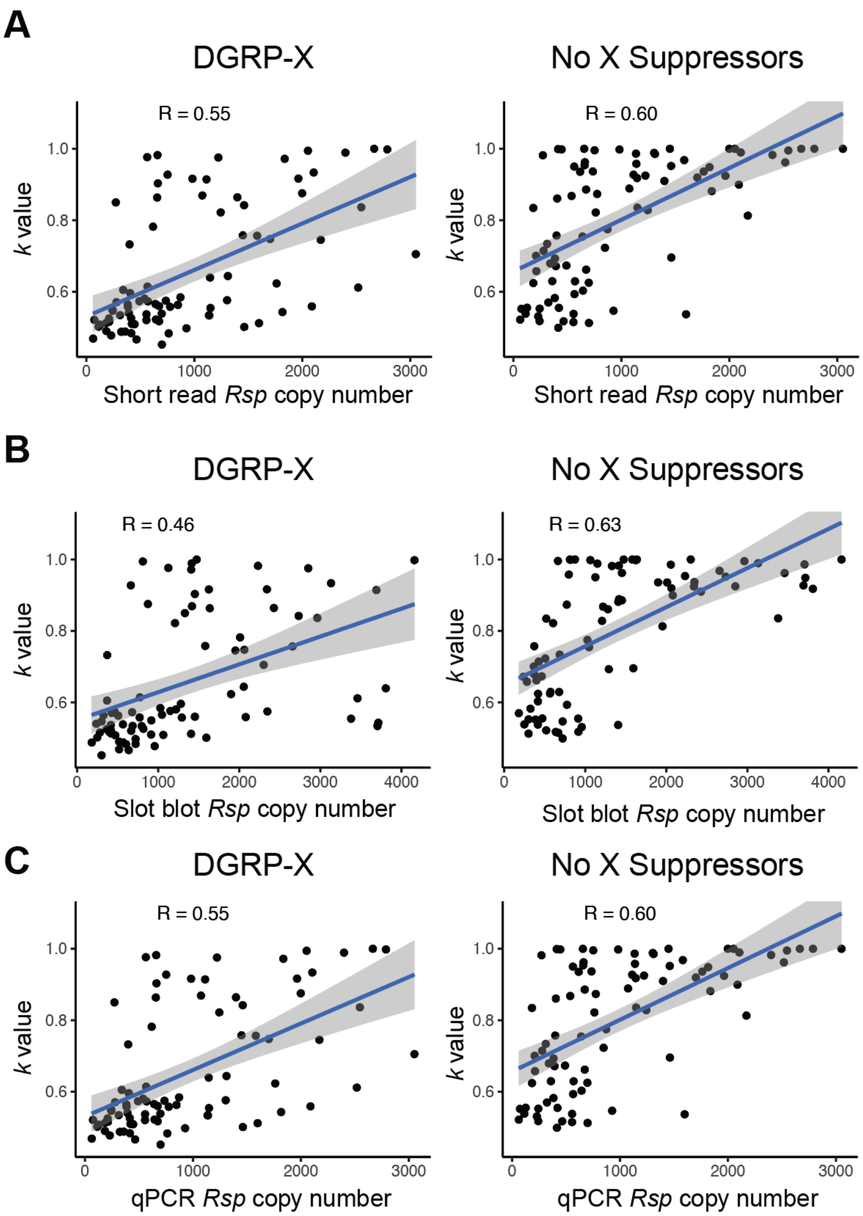

Fig. S2. The positive correlation of *k* values and *Rsp* numbers using different methods across DGRP strains. There is a significant correlation between drive strength (*k* value) and Rsp copy number estimated from either Illumina short read sequence **(A)**, slot blot **(B)**, or qPCR **(C)**. The left panel shows the result from F1 males carrying the X chromosome from DGRP (DGRP-X), and the right panel shows the result from the reciprocal cross (no X suppressors). The high correlation coefficient (R) in each panel suggests that second chromosomes with higher *Rsp* copy are more sensitive to *SD*, even in the presence of suppressors.

bioRxiv preprint doi: https://doi.org/10.1101/2025.10.13.681989; this version posted October 14, 2025. The copyright holder for this preprint (which was not certified by peer review) is the author/funder, who has granted bioRxiv a license to display the preprint in perpetuity. It is made available under aCC-BY 4.0 International license.

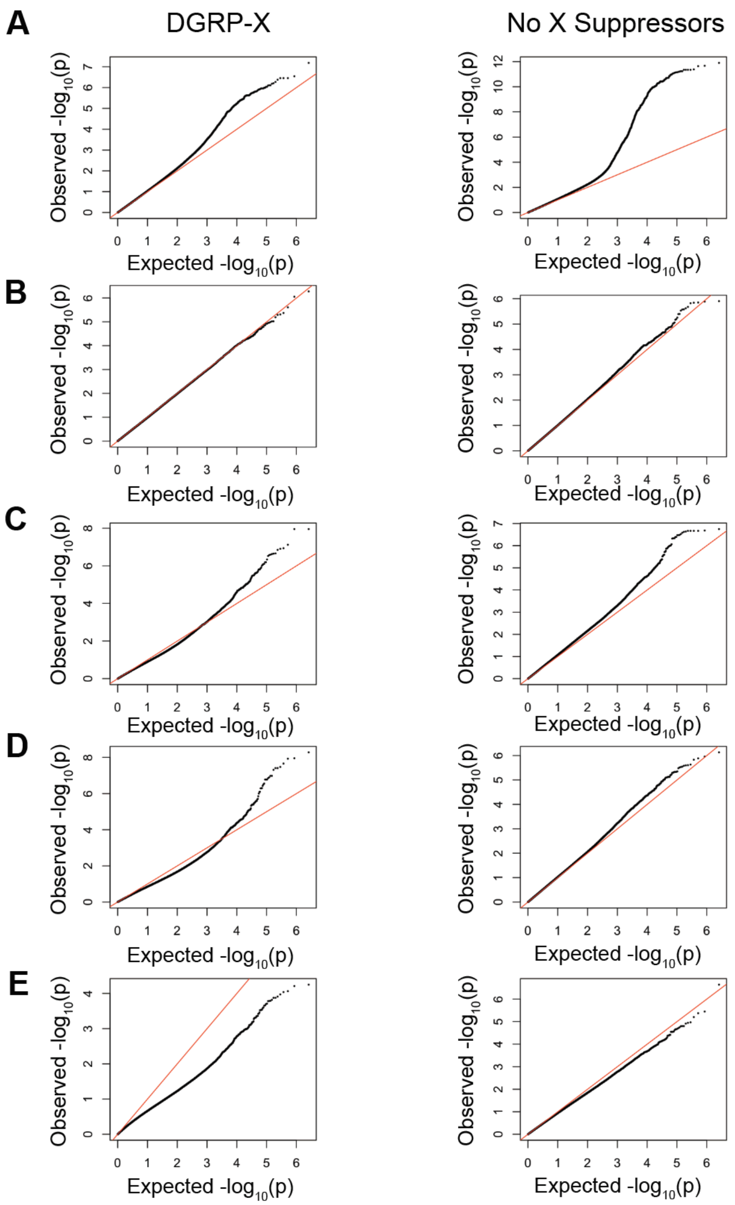

Fig. S3. Quantile-quantile (Q-Q) plots from genome-wide association studies using DGRP strains under different conditions. We generated Q-Q plots to examine the distribution of observed p-values against expected p-values under the null hypothesis for GWAS results. Each row represents using covariates from different methods, including no covariate **(A),** PC1 **(B)**, Short reads **(C),** slot blot **(D),** and qPCR **(E).** The left panel presents the results for the "DGRP-X" crossing and the right panel presents the results from the "No X Suppressors" crossing. The black points represent the observed data, the red line indicates the expected distribution under the null hypothesis (y=x), and deviations from this line suggest potential population stratification or true association signals.

bioRxiv preprint doi: https://doi.org/10.1101/2025.10.13.681989; this version posted October 14, 2025. The copyright holder for this preprint (which was not certified by peer review) is the author/funder, who has granted bioRxiv a license to display the preprint in perpetuity. It is made available under aCC-BY 4.0 International license.

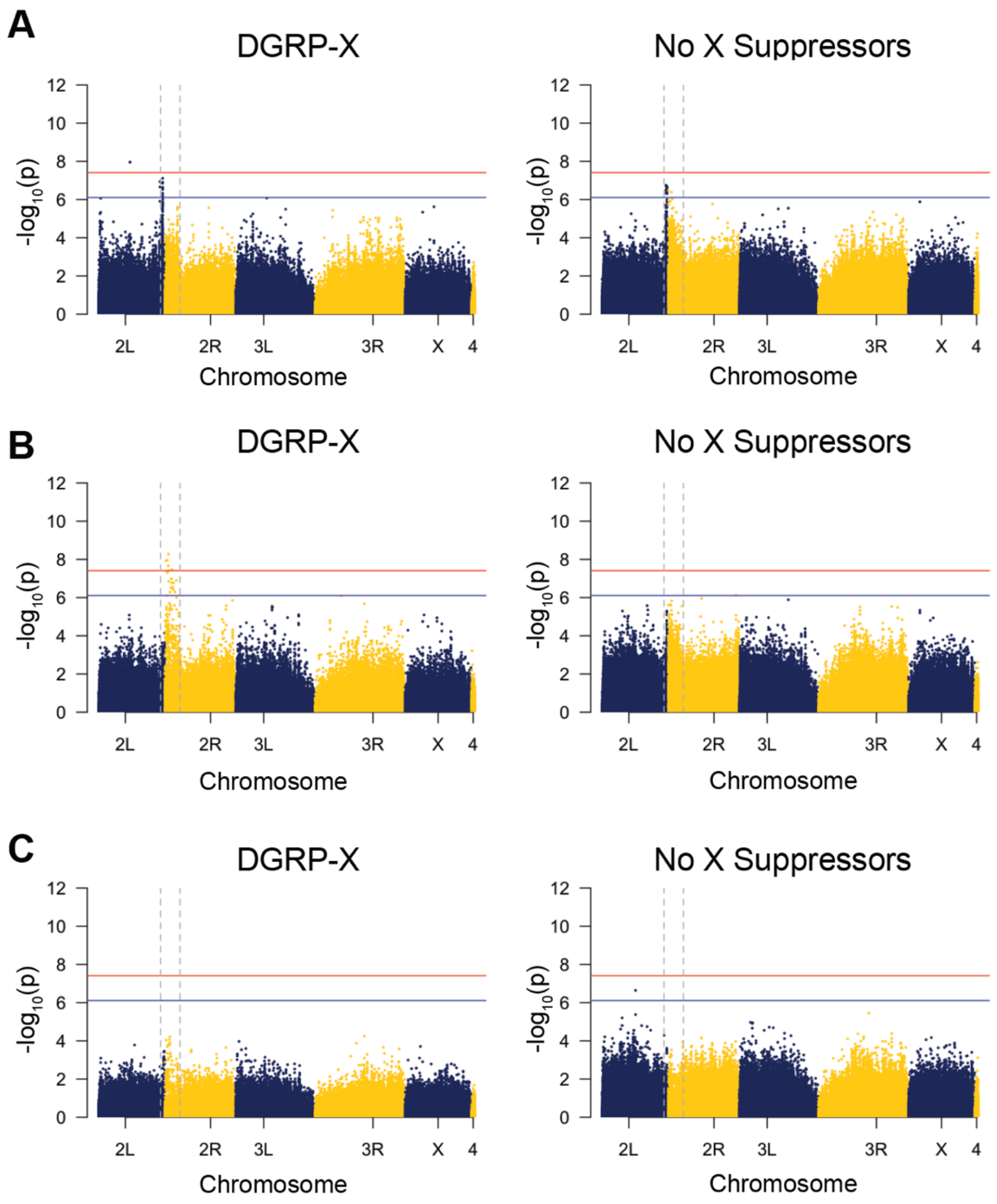

Fig. S4. Manhattan plots of GWAS results for drive strength (*k*) using covariates from different *Rsp* estimation methods. We used *Rsp* copy number estimation from short read **(A)**, slot blot **(B)**, and qPCR **(C)**. Results using *k* from the DGRP-X panel (left) and from the "no X suppressors" (right) are shown, and each dot represents a SNP. The red and blue horizontal lines denote genome-wide significant and suggestive thresholds after Bonferroni's correction.

bioRxiv preprint doi: https://doi.org/10.1101/2025.10.13.681989; this version posted October 14, 2025. The copyright holder for this preprint (which was not certified by peer review) is the author/funder, who has granted bioRxiv a license to display the preprint in perpetuity. It is made available under aCC-BY 4.0 International license.

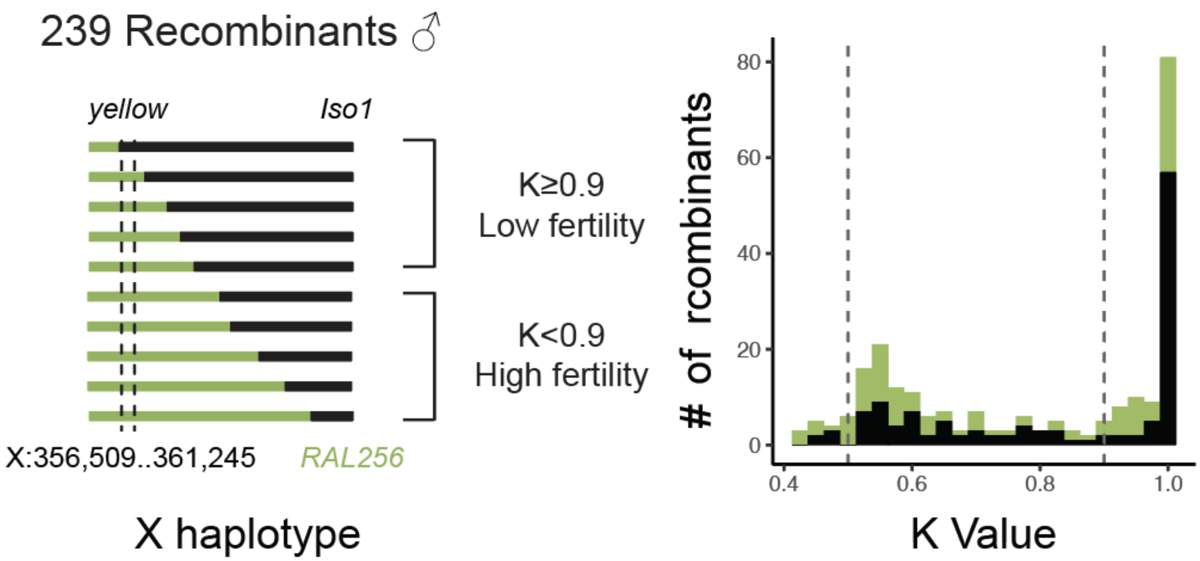

Fig S5. One major locus contributing to the suppressing effect of *Su(SD)X* is not linked with the visible marker *yellow*. We generated 239 recombinants between the *Su(SD)X* from RAL-256 and *yellow* chromosomes. We measured *k* values of these recombinants by counting their offspring. We also genotyped these recombinants to map suppressors. The recombining chromosomes containing *yellow* from the *Su(SD)X* chromosome are marked in green, and those containing *yellow* from the *Iso-1* are marked in black.
